# Editorial: Chronic Liver Disease: New Targets and New Mechanisms, Volume II

**DOI:** 10.3389/fmolb.2023.1237824

**Published:** 2023-07-18

**Authors:** Sofia Jerez, Jinhang Gao, Enis Kostallari

**Affiliations:** ^1^ Division of Gastroenterology and Hepatology, Mayo Clinic, Rochester, MN, China; ^2^ Department of Gastroenterology, West China Hospital, Sichuan University, Chengdu, China

**Keywords:** liver disease, NASH, metabolism, therapeutics, hepatotoxicity

## Introduction

Chronic livers diseases, including non-alcoholic steatohepatitis (NASH), alcohol-related liver disease (ALD) and viral hepatitis, may lead to cirrhosis, which is a leading cause of death (Xu et al., 2022; Devarbhavi et al., 2023). Efficient therapies don’t exist, defining a space of unmet needs. Metabolic pathways play an important role in the establishment of chronic liver diseases, where hepatic stellate cells, immune cells and other cell types play crucial roles (Chen et al. Kostallari and Shah, 2016; Drinane et al., 2017; Maiers et al., 2017; Du et al., 2018; Kostallari et al., 2018; Haak et al., 2019; Hilscher et al., 2019; Arab et al., 2020; Azad et al., 2020; Gao et al., 2020; Mejias et al., 2020; Yaqoob et al., 2020; Kostallari et al., 2021; Greuter et al., 2022; Kostallari et al., 2022; McConnell et al., 2023; Xiao et al., 2023). However, their study is just starting to flourish. This second volume of “*Chronic Liver Disease: New Targets and New Mechanisms*” Research Topic continues to present recent advances in the field of chronic liver disease which might point towards the next potential therapeutic advances.

## Metabolism

The development of metabolomics technologies has enabled the study of metabolic changes in cells and tissues pointing towards pathways that might be involved in the development of liver diseases. Transjugular intrahepatic portal shunt (TIPS) is performed to decrease portal hypertension during liver disease (Kamath and McKusick, 1997). However, TIPS might be associated with increased weight gain and fat mass in patients with cirrhosis (Trotter et al., 1998). To understand how TIPS affects metabolism pathways that could lead to increased fat accumulation, Chen et al. performed metabolomics studies in peripheral and portal serum, before and early after TIPS. They found that in addition to some lipid metabolites that correlated with liver function, metabolism pathways of several amino acids were the main affected ones. In addition, some portal metabolites might be potential predictive biomarkers for liver function decline, even though the results were not statistically significant. In another study, metabolomics studies were performed to study the effect of the Chinese patented medicine, Xuezhiping capsule, on hyperlipidemia and fatty liver in a high-fat diet hamster model. Indeed, Wang et al. demonstrated that Xuezhiping capsule decreased the levels of total cholesterol, triglycerides, low-density lipoprotein cholesterol, increased the levels of high-density lipoprotein cholesterol and alleviated lipid droplet accumulation in the liver of high-fat fed hamsters. However, Xuezhiping capsule increased the biochemical indexes of oxidative stress, which is usually associated with fatty liver disease (Fromenty and Roden, 2023). Thus, further studies are important to deeply assess the beneficial role of the Xuezhiping capsule in fatty liver disease. Another metabolic pathway involved in fatty liver disease is the bile acid (BA) metabolism, which is commented by Bing and Li. Since conversion into BAs is the main way to eliminate cholesterol from the body, dysregulation of this pathway is associated with obesity, non-alcoholic fatty liver disease (NAFLD) and other metabolic diseases. Moreover, total BA levels are elevated, and their composition is changed in the hepatic-intestinal circulation in patients with NASH. All the above studies demonstrate that metabolism can drive liver dysfunction and additional studies are needed to fully understand the role of metabolic pathways and their crosstalk during liver diseases.

## Non-alcoholic fatty liver disease

NAFLD is a public health concern affecting 30% of the world population (Younossi et al., 2023). Progression of the injury can cause non-alcoholic steatohepatitis (NASH), and eventually cirrhosis and hepatocellular carcinoma (Devarbhavi et al., 2023). Early stages of NAFLD are difficult to diagnose by MRI or ultrasound due to the low sensitivity of these techniques. Therefore, finding correlations between early stages of NAFLD and co-morbidities can improve the diagnosis and outcome of the disease. Utilizing a publically available database including a cohort of eleven thousand patients, a positive correlation between diabetic retinopathy and liver fibrosis has been reported Zhang et al. Their findings suggest the use of diabetic retinopathy as a disease progression predictor of NAFLD. Regarding the later stages of the disease, Bing and Li summarize the effect of BA on NASH-liver cancer progression. In this respect, *in vivo* and *in vitro* studies have shown that taurine deoxycholate (TDCA) and glucose deoxycholate (GDCA) activate hepatic stellate cells and promote liver cancer (Xie et al., 2021). NAFLD progression and liver cancer are also affected by macrophage presence Kohlhepp et al. comment on the diversity of macrophage subpopulations and functions and the complicated roles that these cells have on disease progression.

## Therapeutic strategies

Fat deposition in hepatocytes promotes inflammation and oxidative stress within the liver. BA are key players in lipid metabolism and fat accumulation in the liver. Bing and Li comment on the role of BA homeostasis disruption in chronic liver diseases. They summarize the use of several drugs tested in clinical trials that reduce BA levels, either by blocking their synthesis or promoting their excretion. BA receptor agonist obeticholic acid decreases bile acid production, lipid absorption, as well as hepatic steatosis (Younossi et al., 2019). Natural compounds and dietary supplements, such as curcumin and taurine, pose as another alternative treatment for liver disease. Indeed, these components neutralized the oxidative stress in the liver in an acute model of hepatotoxicity in rats Al-Zahrani et al. In addition, curcumin reduced liver fibrosis and improved other clinical parameters. An additional example consists in analyzing the use of a popular supplement in China, prepared with botanical compounds, for the effective treatment of hyperlipidemia, resulting in reduced levels of lipids in the serum and in the liver Wang et al. Another interesting approach to decrease chronic inflammation in the liver is the targeting of the dysregulated immune checkpoints or specific immune cell metabolism (Tacke et al., 2023). Macrophages, including infiltrating monocyte-derived ones and resident Kupffer cells, are the most abundant immune cells in the liver which are significantly increased with injury (Gao et al., 2021). Kohlhepp et al. review the roles that macrophages play during NAFLD and liver cancer. They highlight therapeutic strategies that decrease inflammation by blocking macrophage infiltration or preventing their activation and the release of inflammatory cytokines. All these emerging potential therapeutic possibilities might improve the management and outcomes of liver disease, improving patients’ life quality and, hopefully, reducing the high prevalence rates.

## Conclusion

This second volume of the Research Topic “*Chronic Liver Disease: New Targets and New Mechanisms*” gathers some recent original research studies and reviews on the role of metabolic pathways during fatty liver disease and potential therapeutic approaches. Although novel findings are paving the path to a better future, the understanding of the mechanisms is still uncomplete and requires further studies.
